# Redox Activities and ROS, NO and Phenylpropanoids Production by Axenically Cultured Intact Olive Seedling Roots after Interaction with a Mycorrhizal or a Pathogenic Fungus

**DOI:** 10.1371/journal.pone.0100132

**Published:** 2014-06-26

**Authors:** Francisco Espinosa, Inmaculada Garrido, Alfonso Ortega, Ilda Casimiro, Mª Carmen Álvarez-Tinaut

**Affiliations:** 1 Departamento de Biología Vegetal, Ecología y Ciencias de la Tierra, Universidad de Extremadura, Avenida Elvas s/n, Badajoz, Spain; 2 Departamento de Anatomía, Biología Celular y Zoología, Universidad de Extremadura, Avenida Elvas s/n, Badajoz, Spain; Institute for Plant Protection (IPP), CNR, Italy

## Abstract

Roots of intact olive seedlings, axenically cultured, were alternatively placed in contact with *Rhizophagus irregularis* (mycorrhizal) or *Verticillim dahliae* (pathogenic) fungi. MeJA treatments were also included. *In vivo* redox activities in the apoplast of the intact roots (anion superoxide generation, superoxide dismutase and peroxidase activities) were measured. All our results showed that apoplastic redox activities of intact seedling roots in contact with the compatible mycorrhizal fungus were clearly attenuated in comparison with the pathogenic fungus or treated with MeJA, even at the early stages of treatment used. Total phenolics, flavonoids and phenylpropanoid glycosides were also quantified. Roots in contact with the mycorrhizal fungus did not enhance the biosynthesis of phenolic compounds with respect to controls, while those in contact with the pathogenic one significantly enhanced the biosynthesis of all phenolic fractions measured. Reactive oxygen species and nitric oxid accumulation in roots were examined by fluorescence microscopy. All of them presented much higher accumulation in roots in contact with the pathogenic than with the mycorrhizal fungus. Altogether these results indicate that intact olive seedling roots clearly differentiated between mycorrhizal and pathogenic fungi, attenuating defense reactions against the first to facilitate its establishment, while inducing a strong and sustained defense reaction against the second. Both reactive oxygen and nitrogen species seemed to be involved in these responses from the first moments of contact. However, further investigations are required to clarify the proposed crosstalk between them and their respective roles in these responses since fluorescence images of roots revealed that reactive oxygen species were mainly accumulated in the apoplast (congruently with the measured redox activities in this compartment) while nitric oxid was mainly stored in the cytosol.

## Introduction

Early root defense reactions imply an oxidative burst with a rapid generation of Reactive Oxygen Species (ROS) in the apoplast, as superoxide (O_2_^−^) and hydrogen peroxide (H_2_O_2_) [1],[2]. This is the consequence of activation of plasmamembrane (PM) redox chains such as NADPH oxidase (NOX/RBOH and perhaps other components linked to it), playing a key role the cell wall peroxidases, superoxide dismutases and catalases [3],[4],[5],[6]. ROS are produced as a result of aerobic metabolism or in response to stresses [7], becoming necessary for plant defense during plant-pathogen interactions [1]. Avirulent pathogens induced a biphasic ROS production [8], but in the case of virulent pathogens only the first phase has been detected [9].

Pathogenic fungi extend their hyphae and directly penetrate into the epidermal or mesophyll layers of plant cells, developing specialized structures for the exchange of nutrients such as haustorium [10]. These structures released different effectors to the plant apoplast, as small peptides [11] that act by enhancing the infection and enzymatic activities assigned to suppress the defense response [12]. More recently it has also been proposed participation in this process of ROS released by pathogenic fungi [13]. Plant defense response to pathogenic fungi included changes in pH, ionic fluxes, and production of ROS in the apoplast, this directly involved as toxic compounds, and also in signalling and hypersensitive response. These processes also included the reinforcement and cross-linking of cell walls and cell defense compounds production [4]. The production of high levels of ROS also induced synthesis of antioxidant compounds and detoxifying activities of ROS such as SOD, peroxidases and other antioxidant like phenolics compounds. The production of ROS can be achieved by the action of the RBOH and/or apoplastic peroxidases [4], [14].

The phenilpropanoid metabolism is another defensive mechanism [15]. Phenols play an important role as antioxidant and in the modification of the properties of cell walls, limiting polysaccharide degradation by exogenous enzymes and increasing cell wall rigidity [16]. Some phenylpropanoids can polymerize and form defensive structures, such as lignin [17]. Gayoso et al. [16] concluded that *Verticillium dahliae* infection had a clear influence on phenolic metabolism in tomato, the increase in total phenolics being detected after 2 h inoculation in the resistant lines. So, a higher content of these compounds is indicative that strong defense reactions are being displayed by the plant.

Nitric oxid (NO) is a highly reactive signal molecule, but the origin of NO in plants remains mainly unclear. In the cytosol the Nitrate reductase (NR) catalyzes the reduction of nitrate to nitrite using NADH. The NR-mediated NO production can be induced by biotic or abiotic factors, including elicitors from fungal plant pathogens [18]. More recently, a nitrite: NO reductase (NI-NOR) was discovered in PM from plant roots, involved in NO formation [19]. In plants, NO is involved in morphogenetic and physiological processes including responses to biotic or abiotic stresses [20],[21]. Therefore, NO is involved in plant-pathogen and plant symbioses interactions [22],[23],[24] as well as plant responses induced by elicitors [25]. High concentrations of NO can have a synergistic effect with ROS leading to defense reactions [26]. In the reactions induced by pathogens or their avirulent strains, the O_2_^−^ produced can react with NO to form peroxynitrite, an even more reactive agent to many pathogens. Nevertheless, the role of NO and ROS in symbiotic and pathogen interactions remains unclear.

So, the ability of plants to sense and respond to the attack of fungal pathogens is one of the first events in the evolutionary process of land plants. Linked to this process is interesting to note the capacity developed by plants to establish fungal symbiosis, which demonstrates the ability to differentiate between pathogenic and symbiotic interactions [12]. The involvement of oxidant and antioxidant systems in mycorrhizal symbiosis is well known. For instance, ROS production and activation of NOX/RBOH has been evidenced during mycorrhizal symbiosis [27], while Fester and Hause [28] suggested that ROS play a role in the control of mycorrhizal interactions. Lambais et al. [29] showed an induction of SOD activities in the establishment of arbuscular mycorrhiza (AM), this activation perhaps associated with the high levels of H_2_O_2_ observed in bean roots colonized by *Rhizophagus irregularis*. These authors concluded that the production of H_2_O_2_ by SODs could be associated with fungal recognition and activation of the plant defense. Baptista et al. [30] showed that two of three H_2_O_2_ peaks detected in the early stages of the ectomycorrhizal (EM) interactions between *Castanea sativa* and *Pisolithus tinctorius* (from 2 to 15 h), coincided with O_2_^−^ bursts and an increase in SOD activity. These data suggest that the control of ROS production is crucial for mycorrhizal stablishment, as an excessive level of ROS can result in cell damage involving lipid peroxidation of cell membranes [31]. Besides, the spread of mycelium occurs only in the root cortex, this suggesting that the host plant exerts some kind of control over fungal proliferation, confining it into specific root tissues. Defense reactions, however, which are usually triggered as a plant response to microbial invasion, are only observed in a modulated form in AM tissues [32],[33]. In fact, these reactions occasionally result in a local, weak and transient defense response during the early steps of compatible AM interactions [34]. Host plants usually show a cytological reaction to hyphopodia formation or to the first steps of root colonization [23], this attenuated response disappearing at the late stage of symbioses [35]. Likewise, in onion plants cell wall binding chitinases and peroxidases activity increases during the first steps of AM colonization, reaching the lowest levels at the very early stages of symbioses [36]. Symilarly, in tomato roots colonized by the mycorrhizal fungus (AMF) *Rhizophagus irregularis,* POXs are induced at the begining but suppressed later [28]. Recent studies also show the involvement of NO in the initial establishment of mycorrhiza. Calcagno et al. [24] demostrated the rapid NO accumulation in *Medicago truncata* roots innoculated with the exudate from *Gigaspora margarita* spore cell walls. However, the relationship between ROS and NO during the early stages of *in vivo* interaction still remain unclear.

Other elements of the plant defense response have been described for mycorrhizal roots [37]. For instance, the phenylpropanoid metabolism is activated in typical AM interactions, but to a much lower degree than in plant-pathogen interaction [38]. Likewise, AM-specific alterations in the pattern of anti-oxidative enzymes have been reported [28],[39], matching to the appearance of ROS in arbusculated root cortical cells [26].

Jasmonic acid (JA) and its derivative methyl ester methyl-jasmonate (MeJA) are considered to be an integral part of signal transduction pathways in plant defence responses; so, exogenously applied JA and MeJA were involved in both intra- and interplant signalling [40], and mimicked the effects of elicitors [41]. Sunflower and olive seedling roots induced by MeJA showed significant enhancement in O_2_^−^ generation and POX activity, both inhibited by DPI and Ca^2+^ blockers [5], [6], [14]. Also, the H^+^ and K^+^ fluxes were depressed and transitorily reverted by MeJA [5]. Then, MeJA induces redox defense activities that can be taken as a model to be compared with the same induced by other factors.

*Verticillium dahliae* has been choosen in our experiments as this is a well know pathogenic fungus causing the “Verticillium wilt” due to the infection of the vascular tissue in many species, olive trees included [16]. The fungus is very persistent in the infected field, as it colonizes other plants acting as reservoirs, and also because it develops resistant structures (microsclerotia) capable of surviving for decads in the soil. Considerable losses in productivity are caused by the pathogen that attack to all the olive varieties tested, and is repeatedly described as one of the most dangerous plague to olive orchads [42]. The compatible mycorrhizal fungus used in our experiments was *Rizophagus irregularis*, as it has been demonstrated as compatible with olive plants [43].

In accordance with the above related uncertainties, this study focusses on different aspects of defense reactions displayed by our intact axenic olive seedling roots when put in contact with a pathogenic fungus or a mycorrhizal fungus for few minutes until 24 hours (early interactions). In this context we first measured the oxidative burst (O_2_^−^ generation) and the antioxidant enzymatic activities (SOD and POX) in the intact roots. So, all these redox activities measured correspond to the apoplast of roots under *in vivo* and non-invasive physiological conditions, avoiding cells or root organization disruption. In this way we addequatedly reflect only changes in apoplast, where ROS are synthesized and specifically required for further defense reactions. In a second kind of measures, we observed the localization of ROS and NO by fluorescence in non-fixed whole roots or transverse sections. In all measures before we included MeJA treatments as a model of induced defense reactions in roots, in order to compare it with the same reactions induced by the fungi contact. Finally, we measured different fractions of phenylpropanoids compounds in roots homogenates, in order to follow the biosynthesis of these antioxidant compounds related to the lignin formation for hardening of the cell wall, another well know defense reaction to pathogenesis, and also related to signalling in symbiotic interactions.

## Materials and Methods

### Plant material and culture

Fruits of olive tree (*Olea europaea*, L. cv. Verdial de Badajoz) was collected on the private olive grove “Finca Bercial Olivar-EXAASA” (bercial@frutaria.com), located in Vegas Guadiana, Badajoz (Southwest Spain, 38°51′N, 6°51′W), in Extremadura region, where 267.284 ha are cultivated with olive tree. The recolection does not require specific permission and the field studies did not involve endangered species. Fruits of olive tree were harvested once ripe, pulp removed and stones disinfected and dried at room temperature. Stones were then broken, and axenically cultured isolated embryos from the olive seeds were used to obtain axenic intact seedlings [44]. Embryos were germinated in axenic conditions for 10 days in 24h ligth period, at 25±1°C, on filter paper moistened with 3,845 mg/l GA and 1 mg/l ANA in hermetically sealed Petri dishes according to Garrido et al. [14].

### Fungal cultures

AM monoxenic cultures were established as described by St-Arnaud et al. [45]. A carrot Ri-T DNA transformed root culture was grown together with the arbuscular mycorrhizal fungus (AMF) *Rhizophagus irregularis* (syn. *Glomus intraradices,* DAOM 197198, [46]) (EEZ-45; Estación Experimental del Zaidín, Granada, Spain) in two-compartment Petri dishes. Cultures were initiated in the proximal compartment (carrot root compartment), which contained a minimal culture medium (M medium) gelled with 0.35% (w/v) phytagel [47]. Fungal hyphas, but not roots, were allowed to grow over the plastic barrier to the distal compartment (hyphal compartment), which contained the gelled M medium described above, but without sucrose (M-C medium, [45]). Plates were incubated in the dark at 24°C. Pathogenic fungus (PF) *Verticillium dahliae* Kleb. (EEZ2, from the same institution that AMF used) was cultured on potato dextrose agar medium (PDA) in Petri dishes incubated at 22°C in the dark.

### Experimental design

Intact olive seedling roots were alternatively placed in a monoxenic culture system M-C medium, with the profusely grown but not-sporuled AMF, or with the PF in Petri dishes with PDA medium. For MeJA experiments, intact seedling roots were placed in Petri dishes with fresh M-C medium, without fungal hyphas, and treated with 4 µM MeJA. This treatment was only applied in short-time experiments, since MeJA induced root senescence at longer times.

In every case, measurements were made at different interaction times (5, 10, 15, 30, 45, 60 and 120 min in short-time experiments, and 4, 6, 8, 10, 12 and 24 hours in long-time experiments). Controls were performed for each treatment and interaction time experiment (of the short and long range) with similar roots placed in fresh M-C or PDA medium respectively, without any treatment (MeJA or contact with fungal hyphas).

In order to standarize the results, all measurements at every interaction time were carried out almost simultaneously, with intact olive seedling roots from the same culture batch. Consequently, control, MeJA treatment and AMF or PF hyphal contact were carried out in the same experiment (made at every interaction time), using 10 seedlings in each treatment or control. Every measurement was carried out in triplicate and every experiment (at every interaction time) was wholly repeated at least 5 times. Data were statistically analyzed by the Mann-Whitney U-Test for signification of treatments with respect to controls and among them.

### *In vivo* measurements of apoplastic (EC = exocellular) redox activities

The O_2_^−^ generating activity of intact seedling roots was assayed spectrophotometrically (Shimadzu UV1603) by measuring the oxidation of epinephrine to adrenochrome [14],[48]. The reaction mixture contained 1mM epinephrine in acetate buffer 25 mM, pH 5.0. The reaction was started by immersing the intact roots into the continuously aerated reaction mixture at 25°C [6]. A little volume of the reaction mixture was taken up with the sipper after 30 min and monitored the A_480nm_ (extinction coefficient of 4.020 mM^−1 ^cm^−1^).

The exocellular nonspecific peroxidase (ECPOX) activity of intact roots was assayed spectrophotometrically with the sipper unit according to Ngo and [49]. The reaction mixture contained 3.3 mM 3-dimethylaminobenzoic acid (DMAB), and 66.6 µM 3-methyl-2-benzothiazolinonhydrazon (MBTH) in 100 mM phosphate buffer pH 6.0. A unit of ECPOX is defined as the amount of enzyme required to cause the formation of 1 nmol DMAB-MBTH (indamine dye) per minute at 25°C, pH 6.0. Measures were carried out as above described, but at A_590 nm_ (extinction coefficient of 47.6 mM^−1 ^cm^−1^).

Determination of exocellular superoxide dismutase (ECSOD) activity was carried out according to McCord and Fridovich method [50], based on the inhibition of cytochrome *c* reduction in the presence of xanthine-xanthine oxidase. The reaction was performed in 50 mM phosphate buffer (pH 6.0) containing 0.1 mM EDTA, 0.1 mM xanthine, 1 mM cytochrome *c* and 0.01 mM NaCN. The reaction was started by the adition of xanthine oxidase from an initial solution of 0.06 U/ml to produce an increase in A_550nm_ of 0.025 unit/min. Changes in absorbance were registered after 3 min incubation of the roots in 1.5 ml reaction mixture. Blank reactions were performed using the same mixture without xanthine oxidase. A unit of ECSOD is defined as the amount of enzyme that inhibits cytochrome *c* reduction by 50%.

### Biochemical analysis of phenolic compounds in roots extracts

Total phenolics, flavonoids and phenylpropanoid glycosides were extrated from seedling roots by homogenization with methanol, chloroform and 1% NaCl (1∶1∶0.5). The homogenate was filtered and centrifuged at 5000 *g* for 10 min and the methanolic phase separated, where the phenolic compounds were determined. Total phenolics content was assayed quantitatively by A_765_ with Folin-Ciocalteau reagent [51]. The results obtained were expressed as mg of cafeic acid g^−1^ FW. The total flavonoids content was measured colorimetrically [52] and the total flavonoids content was calculated using the standard rutin curve and expressed as µg of rutin mg^−1^ DW. Phenylpropanoid glycosides (PPGs) were determined by a colorimetric method (A_525_) based on estimating an o-dihydroxycinnamic derivative using the Arnow reagent as described in Gálvez et al. [53]. The concentration was calculated on the basis of the standard curve of 3,4-dihydroxyphenylalanine, and expressed as µg verbascoside g^−1^ FW.

### Images for detection of ROS and NO accumulation in roots

Roots of intact seedlings were alternatively placed in contact with AMF or PF hyphas for 1, 12 or 24 h. Controls were performed with similar roots placed in the medium without fungi. After each treatment, the roots were incubated for 30 min at 37°C (60 min at 25°C for NO detection) in darkness, with 30 µM DCF-DA (for peroxide accumulation), 15 µM DHE (for superoxide accumulation) or 10 µM DAF-2DA (for NO accumulation) in 10 mM Tris-HCl (pH 7.4), and washed twice for 10 min each in the same buffer [54]. After rinsed, whole roots or transverse (non fixed) slices of the different root zones were placed on a microscope slide and examined by fluorescence microscopy (Axioplan-Zeiss microscope). As negative controls, roots were pre-incubated before adding the probes in darkness for 60 min, at 25°C, with 1 mM ascorbate (peroxide scavenger), 1 mM TMP (superoxide scavenger) or 400 µM PTIO (NO scavenger). Parameters for fluorescence microscopy were identical for all experiments and control samples were always included. Images were processed and analyzed using the ImageJ program and fluorescence intensity was expressed as arbitral units (A.U.). At least five roots were tested under each experimental condition and five independent repeats were analyzed.

## Results

### *In vivo* redox activities in the axenic intact seedling roots apoplast

**Figure 1:**
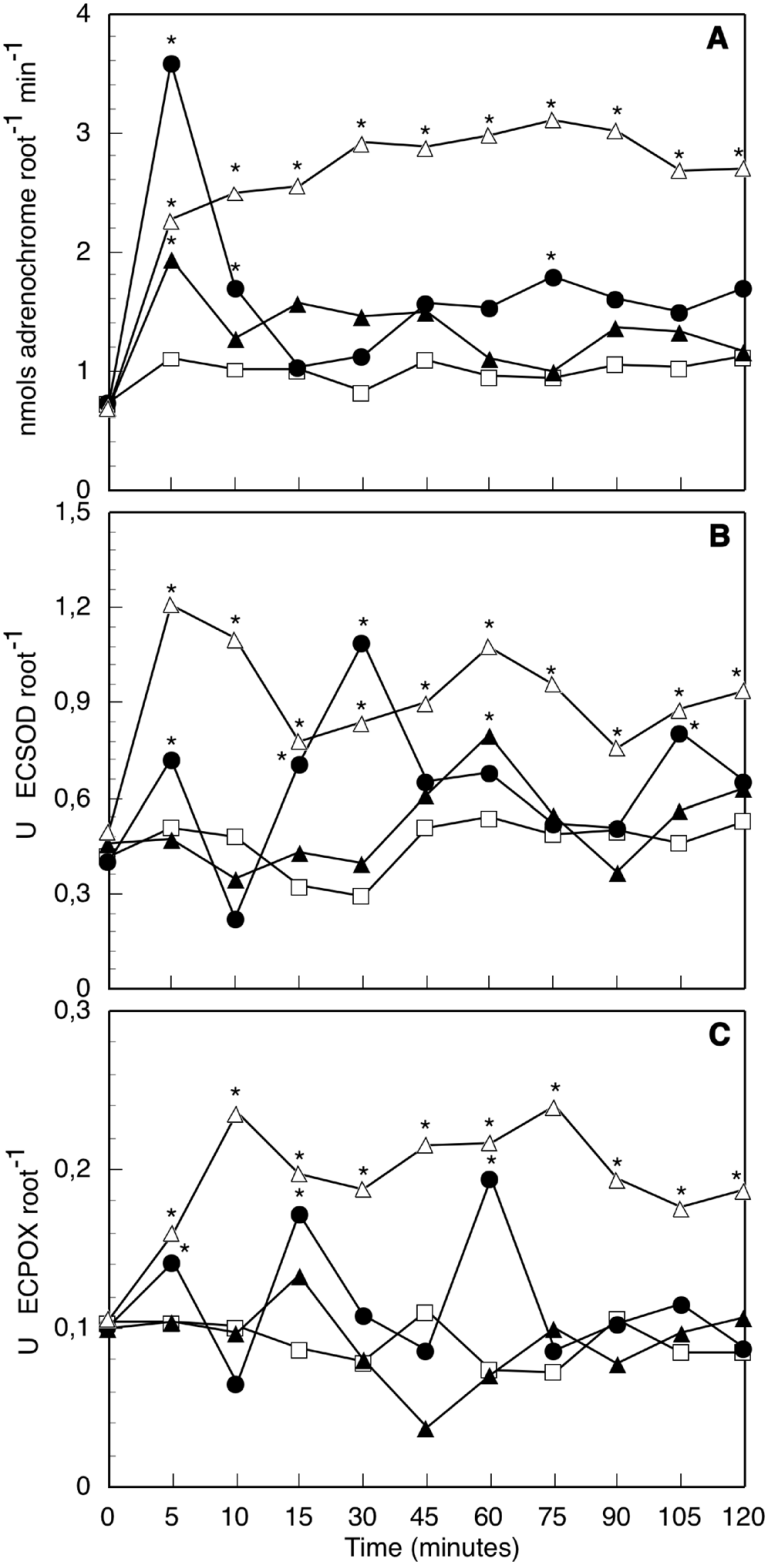
Redox activities measured in short-time experiments. Superoxide generation (A), ECSOD (B) and ECPOX (C) activities in the apoplast of axenic intact olive seedling roots, after 0 to 120 min contact with hyphas of compatible arbuscular mycorrhizal fungus, *Rhizophagus irregularis* (AMF) (-▴-), or the pathogenic fungus, *Verticillium dahliae* (PF) (-▵-), or with methyljasmonate (MeJA) treatment (-•-), in comparison with controls without any treatment (-□-) at the same times. Data from a representative one of 5 independent experiments, each one carried out in triplicate (*significant at p≤0.05, Mann-Whitney U-test).

**Figure 2:**
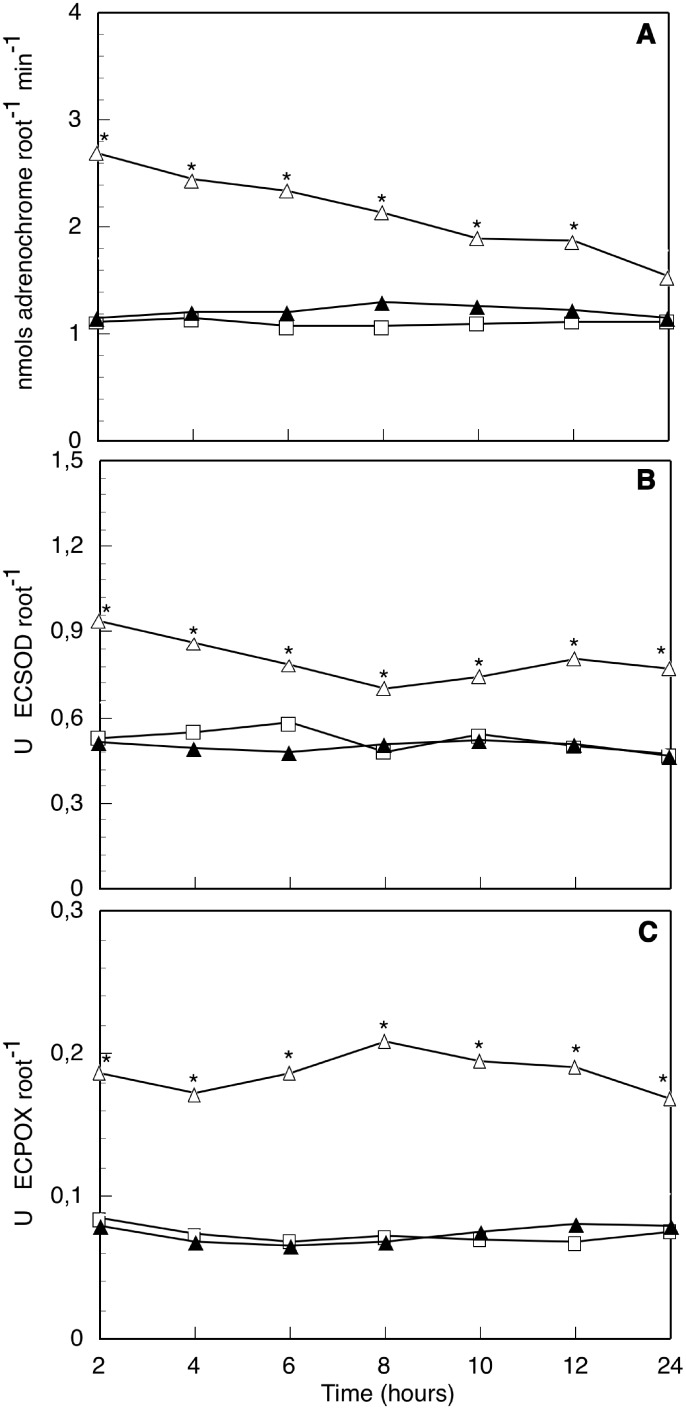
Redox activities measured in long-time experiments. Superoxide generation (A), ECSOD (B) and ECPOX (C) activities in the apoplast of axenic intact olive seedling roots, after 0 to 120 min contact with hyphas of compatible arbuscular mycorrhizal fungus, *Rhizophagus irregularis* (AMF) (-▴-), or the pathogenic fungus, *Verticillium dahliae* (PF) (-▵-), in comparison with controls without any treatment (-□-) at the same times. Data from a representative one of 5 independent experiments, each one carried out in triplicate (*significant at p≤0.05, Mann-Whitney U-test).

We carried out short-time experiments (from 0 to 120 min, Figure 1) and long-time experiments (from 2 to 24 h, Figure 2). In short-time experiments MeJA treatments were applied, but MeJA could not be used at longer times (long-time experiments) when it induced root senescence.

O_2_^−^ generation in the root apoplast measured in short-time experiments (Figure 1A), showed a strong and quick oxidative burst after 5 min MeJA treatment. The O_2_^−^ generation by roots was activated up to 4.9-fold over controls, but the peak decayed to similar values after 15 min. A second increase in O_2_^−^ generation was observed after 45 min, with a statistically significant peak at 75 min, both peaks registering much lower increases than that at 5 min (only 1.9-fold). Roots in contact with AMF hyphas also showed a significant O_2_^−^ generation peak after 5 min contact, but this was considerably lower (1.75-fold) than that induced by MeJA. This peak also decayed to control values after 10 min contact. Then, O_2_^−^ production showed slight oscillations until 120 min contact, but the values were not significantly different to controls. Roots contact with PF induced a strong and maintained O_2_^−^ production (not a peak, as before), starting after 5 min contact and reaching a 3.3-fold maximum, with slight fluctuations across all tested contact times (up to 120 min). Activities were always significantly higher than those obtained with AMF and MeJA, except for the MeJA 5 min peak. In long-time experiments (Figure 2A) O_2_^−^ generation by roots in contact with AMF showed similar values to controls, without no appreciable changes for all tested times (from 2 to 24 h). On the contrary, O_2_^−^ generation by roots in contact with PF progressively decayed from the higher levels observed during the short-time experiments (2.4-fold at 2 h) to similar values to controls at the end of the long-time experiments (1.2-fold at 24 h). Values were always significantly higher than controls or AMF treated roots, except for the last value (24 h contact), which was still higher but not statistically significant.

Antioxidant activities (ECSOD and ECPOX) in roots apoplast in short-time experiments (Figure 1B–C) were determined during the time course of elicitation with MeJA or fungal hyphas contact. Intact seedling root controls showed ECSOD (Figure 1B) activity values between 0.294 (30 min) and 0.580 (2 h) USOD/root. Once again, little homeostatic oscillations were registered. ECSOD activity was significantly higher in MeJA elicited roots with respect to controls, showing three significant activity peaks: at 5, 30 and 105 min (1.4, 3.7 and 1.8-fold, respectively). Roots in contact with AMF hyphas showed similar homeostatic oscillations than controls at all contact times; they only showed a slightly higher but significant peak of activity aftyer 60 min contact (1.5-fold). On the contrary, roots in contact with PF induced a high increase of ECSOD activity. All the values (from 5 to 120 min) were significantly higher than controls or AMF contacted roots, showing oscillations with two maximum peaks: the first one after 5 min fungal contact (2.3-fold) and the second after 60 min contact (2-fold). In long-time experiments (Figure 2B) ECSOD activity of roots in contact with AMF showed similar values to controls and to those observed at the end of the short-time experiments (2 h), all throughout tested time (up to 24 h). Contrastingly, ECSOD activity of roots in contact with PF showed much higher values than controls and AMF contacted roots, although slowly decreasing from 2.4-fold at 2 h up to 1.6-fold at 8 h and 1.4-fold at 24 h.

In short-time experiments, ECPOX activity (Figure 1C) detected in control roots showed slight oscillations, as usual. MeJA elicited roots showed two significant activation peaks with respect to controls: after 15 min treatment (ECPOX activity 2.0-fold) and after 60 min treatment (2.6-fold). The rest of the tested times showed similar values to control activities. Roots in contact with AMF hyphas also showed slight oscillations, but no significantly different from the respective controls were observed. These values were always similar or below those obtained by elicitation with MeJA. Contact with PF induced a strong increase of ECPOX activity, all the values being higher than controls or AMF contacted roots. The response showed two oscillations, with two peaks of maximum activity detected after 10 and 75 min contact (2.3 and 3.3-fold, respectively). In long-time experiments (Figure 2C) ECPOX activity of AMF contacted roots was constant and similar to controls from 2 h to 24 h. Roots in contact with PF always showed higher ECPOX activities than controls or AMF roots. The activity was persistent and sustained, presenting no marked peaks.

### Phenolics content in axenic olive seedling roots

Besides ROS, other elements of defense reactions as phenolic metabolites have been described as involved in the pathogenic and symbiotic interactions between fungi and roots. Consequently, total phenolics content and fractions highly related to defense reactions, flavonoids and phenypropanoid glycosides, were examined. Metabolite contents were measured in homogenates of roots from intact axenic seedling put in contact with the AMF or PF for 2, 8, 12 and 24 h.

**Figure 3:**
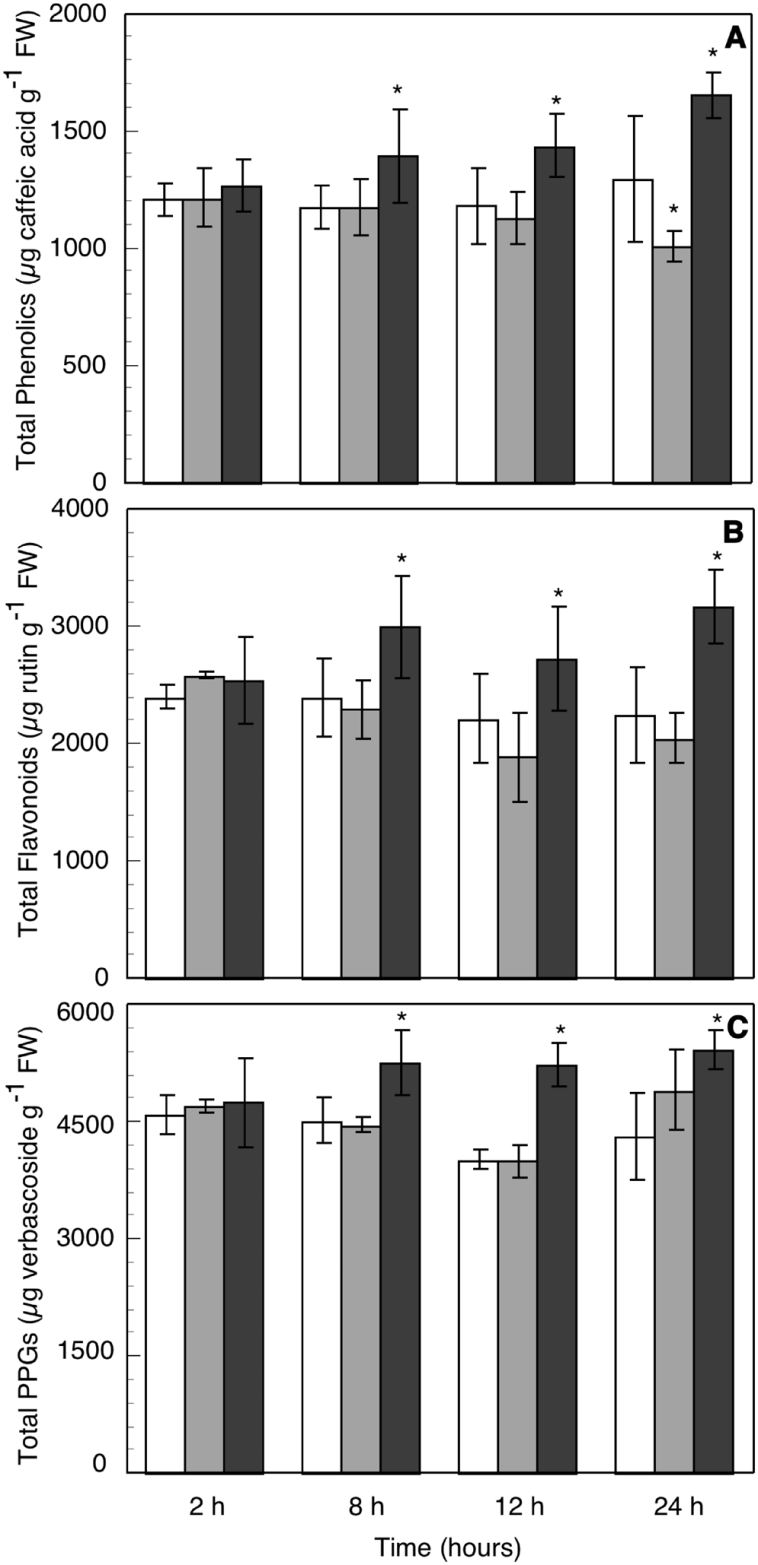
Total phenolics (A), flavonoids (B) and phenylpropanoid glycosides-PPGs- (C) content in extract of roots from intact olive seedling after 2, 8, 12 and 24 h contact with hyphas of compatible arbuscular mycorrhizal fungus, *Rhizophagus irregularis* (AMF) (pale gray bars), or the pathogenic fungus, *Verticillium dahliae* (PF) (dark gray bars), in comparison with controls without any treatment (empty bars) at the same times. Data are means of 5 independent experiments, each one carried out in triplicate (*significant at p≤0.05, Mann-Whitney U-test).

As can be seen in Figure 3, neither of the methabolites contents in control roots significantly changed during all measuring times. Metabolites contents in roots did not significantly change after 2 h contact with any of the fungi, but clear differences between AMF and PF treated roots were observed after 8 h contact. From this time on, total phenolics content (Fig. 3A) in AMF treated roots slowly decayed, although no significant differences were found with respect to controls. On the contrary, at the same times, roots in contact with PF after 8 h showed a steady progressive increase with time in phenolic contents with the maximum value at 24 h (≈31% higher than controls), all values significantly different to controls. A similar response for flavonoids content was observed (Fig. 3B): it significantly increased in PF treated roots with respect to controls from 8 h ahead, with a maximum at 24 h (≈41% higher than controls). Meanwhile, flavonoids content decreased in roots in contact with AMF, although not significantly. A similar response was also obtained for phenylpropanoid glycosides content in PF treated roots (Fig. 3C), where metabolite content was steadily higher than controls in approx. the same extent (26% higher) from 8 to 24 h. Roots in contact with AMF showed values of phenylpropanoids content similar to controls, except at 24 h, when showed intermediate values between controls and PF treated roots.

### Imaging of ROS and NO accumulation in olive roots in contact with MeJA, AMF or PF

**Figure 4:**
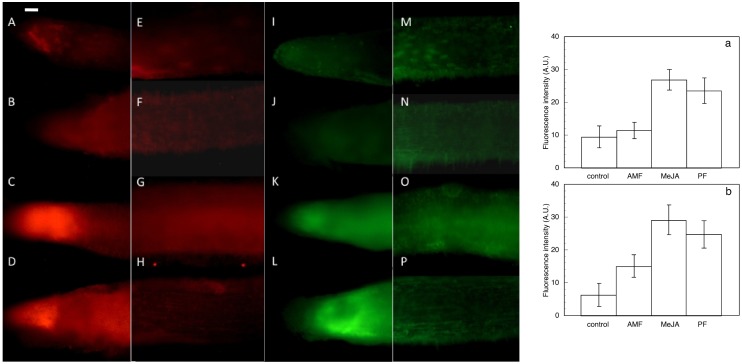
Longitudinal imaging of ROS in olive roots: (A–D: apical zone, E–H: mature zone) imaging of DHE (superoxide anion) and (I–L: apical zone, M–P: madure zone) DCF-DA fluorescence (peroxide) accumulation in olive roots. (A,E,I,M) roots non treated -control-; (B,F,J,N) roots in contact with *Rhizophagus irregularis* 1 h (AMF); (C,G,K,O) roots treated with 4 µM MeJA 1 h; and (D,H,L,P) roots in contact with *Verticillium dahliae* (PF) 1 h. Insert figures show the average fluorescence intensity levels quantified in arbitrary units: a) superoxide-dependent (A**–**H) and b) peroxide-dependent (I**–**P). At least 5 roots were tested for each experimental condition and 5 independent repeats were analyzed. Bar = 100 µm.

**Figure 5:**
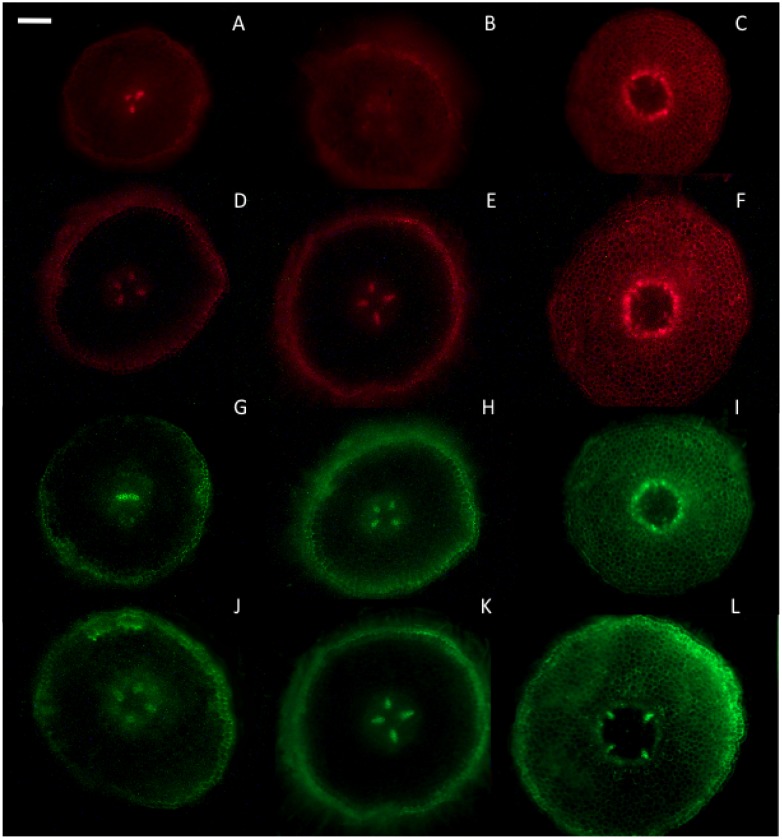
Transversal imaging of ROS in olive roots: (A–C: apical zone, D–F: mature zone) Imaging of DHE (superoxide anion) and (G–I: apical zone, J–L: mature zone) DCF–DA fluorescence (peroxide) accumulation in olive roots. (A,D,G,J) non treated roots -control-; (B,E,H,K) roots in contact with *Rhizophagus irregularis* 24 h (AMF); (C,F,I,L) roots in contact with *Verticillium dahliae* (PF) 1 h. At least 5 roots were tested for each experimental condition and 5 independent repeats were analyzed. Bar = 100 µm.

Figure 4 shows longitudinal images of O_2_^−^ and H_2_O_2_ accumulation in intact olive roots treated with MeJA, AMF and PF, respectively, together with the respective quantitative graphics of fluorescence intensity. In untreated roots (controls) the O_2_^−^ and H_2_O_2_ formation were preferentially localized in the meristematic zone, root cap and vascular cylinder. The fluorescence detected in the elongation zone was weak and restricted to epidermis, hypodermis and vascular tissues. Cortex cells only showed fluorescence in the apex. The fluorescence observed in the apical zone after 1 h and 24 h of fungi contact showed a similar distribution pattern to the above described, for all treatments. However, differences in fluorescence intensity were observed among roots treated with AMF, PF or MeJA. So, longitudinal images of PF and MeJA treated roots showed higher levels of fluorescence than those induced by AMF or untreated roots. In the elongation and basal zones of untreated roots the fluorescence was restricted to epidermal cell and vascular cylinder walls. AMF treated roots showed a similar distribution pattern, but with slightly higher fluorescence intensity. In Fig. 5 transversal sections of the same non-fixed roots were observed. PF and MeJA treated roots showed a much more intense fluorescence which also extended to the cortex cells. Fluorescence was mainly restricted to the cell walls in all cases, although a few cells also showed fluorescence of the cytoplasm in the PF treated roots at 24 h.

**Figure 6:**
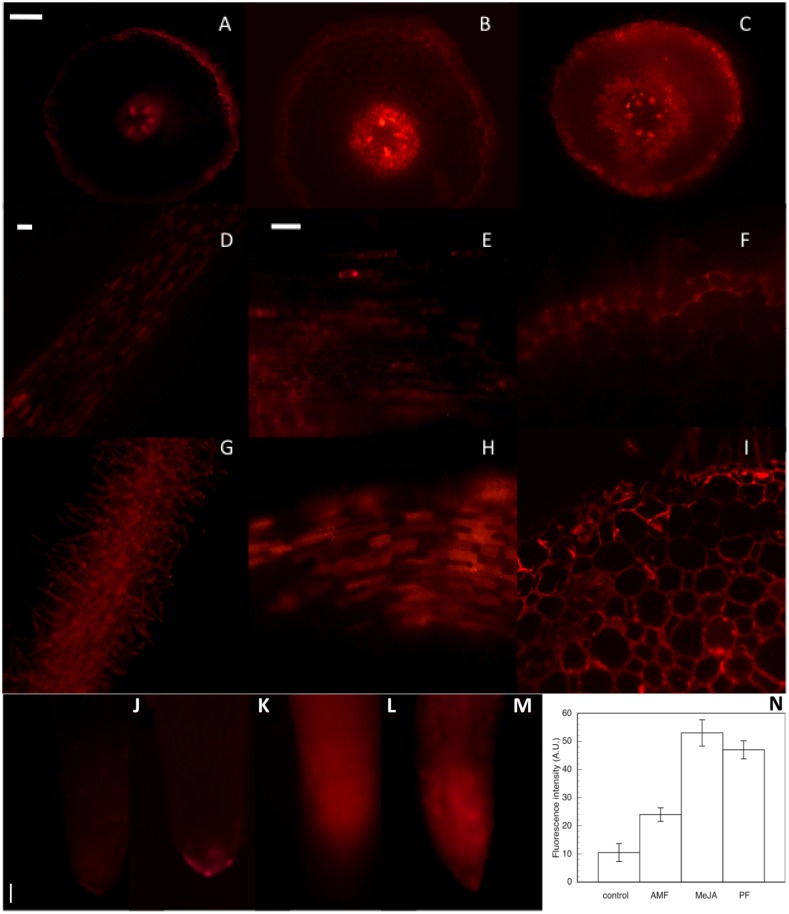
Imaging of NO in olive roots: (A–C: mature zone) Transversal imaging of DAF-2DA fluorescence accumulation in olive roots. (A) non treated roots -control-; (B) roots in contact with *Rhizophagus irregularis* 1 h (AMF); (C) roots in contact with *Verticillium dahliae* (PF) 1 h. (D) Longitudinal imaging of root in contact with AMF; and (G) root in contact with PF. (E,F) Detailed longitudinal and traversal sections of roots in contact with AMF; (H,I) roots in contact with PF showed fluorescence in cytoplasm. (J**–**M) Longitudinal imaging of DAF-2DA fluorescence accumulation in olive roots. (J) roots non treated -control-; (K) roots in contact with *Rhizophagus irregularis* 1 h (AMF); (L) roots treated with 4 µM MeJA 1 h; (M) roots in contact with *Verticillium dahliae* (PF) 1 h; and (N) average fluorescence intensity levels quantified in arbitrary units. At least 5 roots were tested for each experimental condition and 5 independent repeats were analyzed. Bar = 100 µm.

The location and level of NO was analized using the fluorescent probe DAF-2DA-dependent (Fig. 6), togheter with the corresponding quantitative fluorescence intensity are shown. In untreated roots (control) the fluorescence was limited to meristems, vascular cylinder and epidermal cells. In the presence of cPTIO the fluorescence signal was not observed. Roots in contact with AMF for 1 h showed an enhancement in fluorescence of meristems, epidermis and hypodermis, some areas of cortical tissues and vascular cylinders. A fluorescence boost was detected when the roots were placed for 1 h in contact with PF, including cortical cells of the elongation and basal zones. Fluorescence was localized in cell walls but also in cytoplasms. After 1 h contact with AMF or PF, fluorescence enhancements were statistically significant with respect to controls. However, PF treated roots showed stronger fluorescence intensity than roots in contact with AMF, fluorescence extending across cell walls and in the cytoplasm of epidermal and cortical cells, while AMF roots showed fluorescence only in epidermal and hypodermal cells.

## Discussion

With respect to the measurements of the oxidative burst (O_2_^−^ generation) and the related antioxidant activities (ECSOD and ECPOX) we want to emphasize the use of intact seedling roots to measure such redox activities, working under *in planta* non invasive physiological conditions, avoiding cell or root disorganization. Thus, our results specifically reflect the relevance of the oxidative burst and the antioxidant protective enzymes in the root apoplast, without interferences from other cell compartments. The oxidative burst is one of the earliest and more generalized defense reactions evoked in plants by pathogen invasion and many other stresses. The plant defense hormone jasmonate is involved in this defense reaction and specifically required for further ones. We included a MeJA treatment in our experiments as this phytohormone of the defense reactions mimics the effects of pathogen attack, and so we could compare the redox activities evoked by MeJA with those evoked by AMF and PF contact in our roots. Additionally, experimental measurements reported here were timed to allow early detection of AMF interactions with the host plant, as well as differences with PF attack.

We also want to point out that, among the different ECPOX activities tested, we chose the DMAB-MBTH POX as it proved to be competent in measuring POX activity in roots apoplast. Other POX, like guaiacol-POX (ECGPOX), presented activity levels that were too low to allow an accurate discrimination. Catalase activity was also tested since this enzyme also consumes H_2_O_2_, like POX does, but its activity in the apoplast could not be detected as well.

As a consecuence of working under the described *in planta* conditions, the measured *in vivo* redox enzymatic activities of intact seedling roots showed homeostatic oscillations that could be observed to a greater or lesser extent, and were similar among controls and treated roots. In our system, these oscillations presumably maintain a physiological balance in the redox state through the compensation among the systems generating ROS (O_2_^−^) in the oxidative burst, and those detoxificant enzymes induced by them that would eliminate ROS (SOD dismutates O_2_^−^ to H_2_O_2_ and POX uses H_2_O_2_ to oxidize substrates). In any case, an exact match among high levels of O_2_^−^ and low levels of the antioxidant activities, and vice versa could only be observed if all the measurements were carried out within the same root, since there are timing differences from one root to other, as we previously demonstrated with sunflower roots treated with MeJA [5]. Obviously, our present experiments were necessarily carried out with several different roots, although precautions were taken to standardize the results as described in Material and Methods section (at every time all redox activities were almost simultaneously measured with seedling roots from the same batch). Consequently, only approximate matches were generally reached in our results.

The most interesting results were obtained from the short-time experiments, among measures recorded after 5 min to 2 h contact (Figure 1). Within this time interval, roots treated with MeJA showed a very early (5 min) strong peak of O_2_^−^ generation in the oxidative burst evoked by the phytohormone (as we observed in our previous works [5],[14]), followed by approximately successive enhancement peaks of ECSOD and ECPOX that presumably lowered the generated O_2_^−^ to control values. A second O_2_^−^ low peak at 75 min was coincident with very low values of ECSOD and ECPOX activities. A similar, but much more attenuated response was observed in roots in contact with AMF. In this case, an early (5 min) peak of O_2_^−^ was also observed [27], but considerably lower than that produced by MeJA, coincident with ECSOD and ECPOX activities similar to controls. The low oxidative burst presumably induced a late enhancement peak in ECSOD activity at 60 min, again coincident with the lowest levels of generated O_2_^−^. No significant enhancement of ECPOX activity was detected. Finally, roots in contact with PF showed different responses to the ones above described for MeJA and AMF treated roots. First, all three measured activities were strongly enhanced in PF treated roots, always surpassing that of controls and AMF roots. Only some peaks of the MeJA treated roots were higher. Secondly, only the early peaks of ECSOD after 5 min and ECPOX after 10 min contact were coincident with the still not too high values of generated O_2_^−^ (at 5, 10 and 15 min). Afterwards, O_2_^−^ generation was steadily maintained very high, without peaks or oscillations, disregarding changes in the antioxidant enzymes (peaks of ECSOD at 60 min and ECPOX at 75 min). A similar response of PF treated roots was also observed in long-time experiments: O_2_^−^ generation was significantly higher than controls, but slowly and constantly decayed from 2 h on, reaching similar values to controls at 24 h, but so ECSOD as ECPOX stayed higher than controls from 2 to 24 h, not reflecting O_2_^−^ oscillations during this time interval. On the contrary, AMF roots maintained similar activities to controls in all cases.

As above mentioned, MeJA experiments were carried out for comparative reasons, since this phytohormone evokes in roots an oxidative burst physiologically controlled by antioxidant activities counteracting O_2_^−^ peaks through homeostatic oscillations that restore the redox balance of the system, as above described for MeJA and AMF roots. This response to MeJA has been studied in our previous work with sunflower and olive roots [5],[14]. On the contrary, roots contact with the PF used in our experiments (*Verticillium dahliae*) evoked a strong redox defense reaction, characterized by an early but anomalous enhancement of O_2_^−^ generation, which was not a peak but a steady and constantly high level of O_2_^−^. This reaction was not counteracted by the also very high and oscillating levels of ECSOD and ECPOX, since these oscillations were not reflected in changes in O_2_^−^ generation. The latter only slow and constantly decayed from 2 to 24 h, although in this interval the antioxidant enzymes again showed very high and oscillating values during this time interval. This response could be qualified as “non physiological” (since the redox balance was not quickly restored, as was the case after MeJA treatment or AMF contact), and was also different to common oxidative bursts described as oxidative defense reactions evoked by pathogens attack to other plants, similar to that induced by MeJA. According to our results of fluorescence images, in which we showed a much higher production of NO by PF treated roots (see below) we can propose a suggestive hypothesis. Perhaps this anomalous response could be related to the effect of the toxin produced by this PF (*V. dahliae*), which has been described to induce a strong and continual generation of NO in plants [18]. Since NO has been reported to modulate the PM NOX-RBOH activity [26], this being the main source of O_2_^−^ generation in roots apoplast, the PF toxin could be provoking the high and continuous generation of O_2_^−^, thus maintaining the oxidative burst throughout the measuring interval, and preventing it to be physiologically controlled by the antioxidant enzymes. We have an example of such a way of action in plants: fusicoccin, a phungal toxin, continuously and largely enhances PM H^+^-ATPase activity, preventing it to respond to physiological controls. The described special oxidative response of olive roots to the PF *Verticillium dahliae* would explain the high sensitivity of the plant to this fungus, whose attack is very quick and effective, causing serious damages to olive plantations [16].

As with typical responses to pathogens, the oxidative burst of O_2_^−^ was dismutated to H_2_O_2_ by ECSOD to reduce its citotoxic effect, but also because H_2_O_2_ in the apoplast can act directly against the pathogen and as a second messenger penetrate in the cell to evoke a signaling cascade inducing medium-long term defense programs. Morever, ECPOX plays a main role in using H_2_O_2_ to oxidize other substrates, such as cell wall phenolic compounds to lignins and suberins, these hardening the cell wall to act as defense barriers against the infection. However, some pathogenic fungi have been reported to need ROS and NO that were required for pathogenesis [26], and even some of them produce ROS and NO by themselves [55],[56]. Perhaps *Verticillium dahliae* could be one of these fungi needing ROS for pathogenesis and then it would induce a continuous oxidative burst with its toxin to drive and favor the infection.

The response of roots to AMF contact was also an oxidative burst, although much more attenuated in all the activities measured. Our results are in agreement with Dumas-Gaudot et al. [32] and García-Garrido and Ocampo [33], who reported a low and transitory O_2_^−^ generation by roots in contact with AMF. Ruiz-Lozano et al. [57] also described a late activation of ECSOD in lettuce roots, although measures were taken when roots were already colonized by *Rhizophagus irregularis* (i.e. 1–3 months from the first contact with AMF) and perhaps cannot be compared with our system. These and other references (see Introduction) conclude that AMF contact with different species roots evoke an attenuated, local, slight and transitory redox defense reaction. In accordance with this, roots treated with AMF hyphas in our study showed low levels of O_2_^−^ generation and ECSOD and ECPOX activities, indicating this fungus is recognized as compatible to olive roots and, consequently, the initial response is getting modulated and attenuated to allow symbiosis establishment. In fact, mycorrhizal fungi apparently fail to unleash a complete unspecific defense cascade by host root. According to Dumas-Gaudot et al. [32], this behavior could be explained not only because the fungus shows low elicitation ability, but also because it possesses fungal inhibitors or because compatibility fungal factors exist which could interact with elicitor’s activity, stopping or reducing it. As can be seen in short-time experiments, the initial defense reaction, characterized by an early oxidative burst, was really attenuated in roots in contact with AMF, never reaching MeJA elicited roots levels (which imitate the pathogenic elicitor’s effect) neither PF treated roots (whose response was higher and steadier than that of MeJA.

It is interesting to emphasize that most of the reviewed references carried out enzymatic activity determinations in roots (including oxidative burst) one to several days or weeks after the interaction with AMF, as one to three months is the normally agreed time to develop a complete mycorrhization process from the first contacts for colonization. Therefore, this study’s interesting and revealing experimental setting is able to reflect what happens from the very first step of recognition on. So, it was during these early stages when the first defense reactions against pathogens attack by ROS production were recorded, and thus was possible to check how roots attenuated these defenses when they were in contact with an AMF, not with a PF.

Taken together, our results regarding redox activities indicate that, contrastingly to what happened with PF, roots attenuated their redox defense reactions against AMF from the very early stages of fungal contact. These results are in agreement with Hause and Fester’s [37] revision on plant-micorrhyzal fungus interactions, where they concluded that defense processes occur in a moderate way after these contacts.

Our phenolic content results also indicate that roots clearly discriminated between AMF and PF contact regarding the biosynthesis of the measured secondary phenolic methabolites (total phenolics and flavonoids and PPGs fractions), these being higher when in contact with PF but not AMF, at least from 8 to 24 h. Induction of phenolic compounds biosynthesis is a classic defense reaction to pathogens attack. Some of these compounds are excreted and linked to cell walls where oxidyzed by ECPOXs with H_2_O_2_ from the oxidative burst, forming lignin and suberin which harden cell walls and avoid or at least difficult pathogen penetration [58]. Others, as flavonoids and some phenylpropanoids, are phytoalexins that plants synthesize as toxins against microorganisms. Therefore, root response to contact with PF could be a typical defense reaction, as all the secondary phenolic methabolites increased with fungal contact time. However, this response was strongly attenuated when roots contacted AMF, and no significant increases in phenolics biosynthesis (except a slight increase of PPGs at 24 h) was observed. Meanwhile, it is known that some flavonoids present in roots exudates stimulate micorrhyzation by inducing germination of AMF spores and positively affecting hyphal growth during symbiosis [23],[59]. Also, Vierheiling and Piche [60] observed a slight increase in flavonoids content after 2 h AMF contact with roots with respect to controls. More recently, Abdel-Lateif et al. [61] have reviewed the role of flavonoids in the establishment of plant roots endosymbioses, concluding that changes in flavonoid pattern of AMF treated roots suggest they play a regulatory role in first stages of colonization and in later stages of AM association. They literally mention the “flavonoid pattern”, which not necessarily means an increase in total content, but a different composition of flavonoids synthesized by roots, as apparently specific types of these molecules are involved in the interaction. In this study, total flavonoids content did not quantitatively varied in AMF treated roots, but the qualitative pattern of flavonoids might have varied. We are now trying to analyze flavonoids composition of our roots in contact with AMF and PF.

With respect to fluorescence images, in a previous work on olive seedling roots [14] using fluorescence probes DHE and DCF-DA, we showed high levels of O_2_^−^ and H_2_O_2_ generated by untreated roots in epidermis cell walls and the vascular cylinder, both presumably related to differentiation processes [14],[62]. After treatment with MeJA or PF, ROS generation in cortex cells was enhanced [14],[63] in comparison to control and AMF roots. These results are congruent with the above described redox activities in the apoplast, and show the onset of a strong defense response induced by MeJA and PF, while AMF roots presented lower levels of ROS generation and redox activities. Moreover, it is also interesting to point out that in the early steps of AMF contact with roots, ROS generation was restricted to epidermal and vascular tissues, but not to cortical cells. This suggest a role for ROS in driving fungal colonization in the later tissue, as suggested by Dumas-Gaudot et al. [32] and García-Garrido and Ocampo [33]. Sumarizing, our results show that both ROS were strongly generated by roots treated with MeJA or PF, but to a much lesser extent by AMF treated roots. They also show that ROS generation, coincident with redox activities, was restricted to the apoplast at least during the first hours. Later on (24 h), other cytoplasmatic compartments could also begin to be involved in roots treated with PF.

With respect to NO production, our data on root response to contact with AMF are in accordance with those recorded by Calcagno et al. [24] for *M. truncata* roots treated with purified exudate from AMF. The registered lack of NO accumulation in *M. truncata* was faster (5 min) than in our case (1 h), which could be explained by the difference in NO inductor used: AMF hyphas/roots in our study instead of purified exudate. In turn, PF induced a significantly higher and more extensive NO production than AMF. The increased production of NO in response to PF was similar to that described by Shi and Li [18] for Arabidopsis leaves, induced by toxins derived from *Verticillium* and mainly due to the NR pathway. This suggests that roots modulated their response to fungal contact: they responded to AMF signals by inducing an accumulation of NO [24] which was nevertheless lower than with pathogen interactions, when NO levels were much higher, as was observed in tobacco cells treated with the elicitor cryptogein [63]. NO is an important regulator molecule in many physiological processes, especially in response to stress [20],[21], including the plant-pathogen interaction. The role of NO could be the key to the symbiosis establishment and the defense response to pathogenic attacks. The NO production may be linked to cell walls remodeling during early stages of AMF interactions [64],[65], as a novel component of the AM signaling pathway [24]. In pathogenic interactions, NO production may be linked to the plant defense, including the modification of cells and cross-talk with ROS signaling pathway to increase defense reactions [63].

Nevertheless, our results from the earliest measures (obtained from intact roots), showed that NO accumulation was always localized mainly in the cytosol while ROS accumulated in the apoplast (we also did some measures of NO and ROS in the first 15 min, but data are not included). This proved that ROS and NO were not initially produced in the same cell compartments. Thus, according to our results, the oxidative burst located in the apoplast was the earliest, most direct, and relevant defense reaction in biotic interactions [30],[66]. The proposed interactions between ROS and NO are not yet clarified, but when doing so, their different cell localization must be taken into account [24].

In summary, our results indicate that from the earliest stages of contact with AMF, intact roots attenuated the oxidative burst in the apoplast (ROS accumulation and redox activities) in comparison to roots in contact with PF. Congruently, roots in contact with AMF did not enhance the biosynthesis of phenolic compounds with respect to controls [23],[35], while those in contact with PF significantly enhanced the biosynthesis of all phenolic fractions measured. Both ROS and NO were largely accumulated in roots in contact with PF, and much lesser in AMF treated roots. In conclusion, these results proved that intact olive roots clearly differentiated between mycorrhizal and pathogenic fungi, attenuating the defense reactions against AMF to facilitate the arbuscular mycorrhizal establishment, while inducing a strong and sustained defense reaction against PF. Both ROS and NO seemed to be involved in these responses from the first contact moments. However, further investigations must be conducted to clarify the proposed ROS-NO crosstalk and their respective roles in these responses, as roots fluorescence images revealed ROS was mainly accumulated in the apoplast (congruently with the measured redox activities in this compartment) while NO was mainly stored in the cytosol.
